# Routine Manipulations and Operations from the Stand-Point of Asepsis and Antisepsis1Read at the Thirty-sixth Annual Meeting of the American Veterinary Medical Association, September, 1899.

**Published:** 1900-01

**Authors:** Wm. Herbert Lowe

**Affiliations:** Paterson, N. J.; Formerly United States Veterinary Officer of the Port of New York


／# THE JOURNAL
OF
### COMPARATIVE MEDICINE AND VETERINARY ARCHIVES.

VOL. XXI. *JANUARY, 1900.* No. 1.

### ROUTINE MANIPULATIONS AND OPERATIONS FROM THE STAND-POINT OF ASEPSIS AND ANTISEPSIS.[1]

BY WM. HERBERT LOWE, D.V.S.,
PATERSON, N. J.
FORMERLY UNITED STATES VETERINARY OFFICER OF THE PORT OF NEW YORK.

IT is certainly a very nice thing to treat a rare disease or to perform some unusual operation, but the success of the average veterinary practitioner depends far more, in my opinion, upon the personal adaptability, the power of observation, introspection, the methods, *modus operandi*, experience, judgment, and dexterity exercised in the routine manipulations and operations of his practice than in the occasional performance of some rare and unusual operation. Too much importance cannot be laid upon intelligent and thorough attention to method and detail in our everyday practice. The principles underlying all surgical procedures are the same, whether the case be the treatment of an ordinary wound or the performance of one of the more difficult and delicate operations known to veterinary science.

Some people are always waiting for an opportunity to do some wonderful thing, and let thousands of little matters go by that, taken advantage of, would have been productive of surprising results. Success comes to the great majority of people by being able and doing ordinary and common things well. The same is true of the surgeon. Compare the number of times you make an examination or dress an ordinary sore or wound to the number of times you are called upon to perform one of the major surgical operations, and you will, I think, agree with me that the routine

[1] Read at the Thirty-sixth Annual Meeting of the American Veterinary Medical Association, September, 1899.

174615

manipulations and operations are worthy of our consideration at this time and in this place.

More attention should be given in our veterinary schools to the training of students in the art as well as the science, and we would not see graduates of the best schools, who are well educated, not able to diagnose and to put into practice what they have been taught, and making mistakes that are evident to laymen, to say nothing of good horsemen.

In the first place, a man should not study veterinary medicine unless he is naturally adapted to the profession. I sometimes see practitioners who, I think to myself, ought to be behind the counter selling calico instead of practising veterinary medicine. The fact is that a man, in order to make a good veterinarian, should understand the habits, functions, nature, and disposition of different animals, how to go around them, to handle, restrain, and control them. He should be a judge of a horse, and a good horseman in the higher sense of the term.

If he has not the natural adaptability and these acquirements he cannot be considered a qualified veterinarian. I do not care how much scientific and other knowledge he may have, or from what college or university he may have graduated, he is out of his element, and cannot successfully cope with the man with natural intuition and characteristics, other things being equal.

There are many difficulties that are constantly confronting the veterinary practitioner that are not met with in the practice of human medicine. Our patients cannot tell us, *in articulate language*, anything. This fact is often referred to by laymen as well as by professional men. They tell us a good deal, however, if we are sufficiently observant and experienced to understand their language. Every attitude, every position of head, body, and limb, and every movement has a significance, and tells to the trained veterinarian the nature and condition of his patient with just as much certainty, yes, often more certainty and accuracy, than the human being tells his physician of his symptoms and ailments.

Another difficulty is that the commercial value of each patient is, as a rule, taken into consideration in determining whether the animal is to have treatment or not, and if treatment to what extent and cost, his commercial value, rather than his necessity, being the chief factor in determining the matter, whereas, in human practice, no commercial value is ever placed upon a patient.

Another difficulty, and the one that should receive the most attention in the consideration of routine manifestations and operations, is the importance and necessity of aseptic and antiseptic measures in all surgical procedures and dressings. In human medicine, again, our brethren have a decided advantage, so far as aseptic and antiseptic precautions and measures are concerned. The principles involved are essentially the same, but their application is far more difficult, and sometimes impossible in veterinary surgery, as the means available to the veterinary surgeon for satisfying the demands of these principles are not equal to those in human practice. Purification of the air where the animal is kept, complete sterilization and prevention of infection, position, rest, and immobilization in our patients, are problems that to a large extent remain to be solved.

Herbert Spencer says that knowledge is useful in proportion to its application. Aseptic and antiseptic principles of science are of real value to the veterinary practitioner only to the extent that he is able and painstaking enough to put these principles into his everyday practice. It is a comparatively easy thing for any modernly educated veterinarian to outline the principles and practice of aseptic and antiseptic surgery, and as a practitioner he may have the disposition, but he will not always have the opportunity and ability to effect complete sterilization of everything on and about his patient.

The chief question at the present time is how to most perfectly bring practice into accord with knowledge, so as to secure the best results in the ever-varying and widely different conditions in our routine work. It is my opinion that it is in the application of scientific facts and doctrines now well understood that the chief achievements in the near future are to be expected. In the past most all, if not all, the attention and credit were given to the skill displayed in the operating and mechanical part, with little or no intelligent care and after-treatment. Fully as much attention should be given to aseptic and antiseptic precautions and methods, for the science of bacteriology has taught us the relations of microörganisms to the disturbances of wound healing and the importance of preventing infection in simple as well as in extensive wounds; therefore, asepsis and antisepsis is at the very foundation of all routine manipulations and operations, if science is the handmaid of our art.

Aseptic wounds include all which are preserved from contamination or infection of any kind. An aseptic condition in a wound may be obtained either by the protection the wound received from the first against the access of any septic agent, or by the power of living tissues to resist and destroy septic agents, or by the application to the wound of substances which destroy them. As long as asepsis is maintained no decomposition of the secretions or tissues takes place, no sloughing of killed or partly killed tissue occurs. When the proper cares to favor the nutrition of the wounded tissue are rendered the healing of the wound progresses without pain, inflammation, or suppuration, and the least possible amount of cicatrical tissue is produced. To secure an aseptic condition, or to approach it as nearly as possible, is the first and most important indication in all our surgical work and treatments.

Septic wounds include all in which any agent capable of exciting tissue irritation and cell necrosis lodges and grows. They may present the most widely different degrees of wound disturbances dependent upon the varying conditions which the special wound may present and upon the character of the treatment which is instituted, but in all cases they are attended with some degree of inflammation and suppuration and with sloughing of dead tissue. The septic agent may be introduced by the body that inflicts the wound, or by the dressings that are applied, or may be among the dust particles that float in the air to which it is exposed. In very rare cases it is possible that it may be conveyed to the wound through the blood of the animal sustaining the wound.

There are many modifying influences among animals as to their individual ability to recover from injury or surgical interference. Of animals in apparently good physique and enjoying the same hygienic surroundings and treatment, one will recover from a serious injury or surgical operation speedily and without serious complication, while in the other an injury, apparently much less severe, may end fatally or in prolonged illness.

The power of resisting the effects of extraneous influence to some degree is a characteristic of all living matter. Its cessation is death. The quality of the vital resisting power inherent in the constitution of an animal cannot be estimated by any known signs. It may be modified by other conditions, but in some degree it is always present as a powerful unknown factor influencing the result of any case.

Mental impression, shock from injury, old age, and pre-existing organic disease are factors that influence the result of treatment.

There are constitutional differences, such as plethora, anæmia, obesity, that have to be taken into consideration by the practitioner. The temperament, the breed, œstrum, pregnancy, season of the year, faulty hygienic and sanitary conditions, previous overfeeding, previous underfeeding, exhaustion from overwork, exhaustion from physical strain, deleterious effects of vicious habits; these are some of the more marked examples of influences which, by their effect upon bodily nutrition in general, aggravate and affect the result of all routine manipulations and operations.

Good hygienic conditions, by their relations to the functions of nutrition, exert important influences upon all cases. The stimulating effects of sunlight upon nutrition should be regarded in hygienic management. Next to the necessity of fresh air supply, that of sunlight should be provided in arrangement of hospital wards. There is an instinctive craving for the light innate in all living creatures, which becomes more marked whenever, from any reason, there is a depression of vital power.

Insufficient air is synonymous with impure air, for the purest air, if not renewed with sufficient frequency, becomes speedily contaminated by the exhalations and effete materials from the animals breathing it. This, which is true of those in health, is still more quickly accomplished when the animal is diseased. It becomes, therefore, more important for the well-doing of the sick animal than for the welfare of the well that an unlimited supply of pure air should be provided. When to the natural sources of air-contamination there are added the emanations of suppurating wounds, the need of constant change in the surrounding air is more emphatic still if its purity is to be preserved. While air is the great oxygen-carrier for the needs of the living body, it receives in exchange from the body the débris of its disintegration. It is the vehicle of transportation of an infinite variety of floating matter, the great mass of which is organic in character. Putrescible organic matter cannot long be exposed to the air without becoming the recipient of putrefactive germs from it. Aseptic wounded surfaces quickly become septic when exposed by reason of the floating septic particles that are conveyed. The best stimulant to the vital resisting power of a living tissue, by which the effects of sepsis are antagonized and overcome, is perfectly oxygenized blood. The air thus carries both the bane and the antidote. The practical end, therefore, to be aimed at in any given air-supply is that there shall be as small a proportion of the bane and as large a proportion of the antidote as possible. This involves the removal, the suppression, or the diffusion, as much as possible, of all sources of contamination, and the dilution of that which is unavoidable by the introduction of the largest quantity practicable of the purest air attainable. The purity and the sufficiency of the air are thus seen to have a double relation to the healing of wounds, one in a general relation, which the air shares with other hygienic conditions, and the other a special relation as a carrier of and an antidote to sepsis. The hygienic conditions in the wounded animal must be made as good as possible before the whole duty of the surgeon is accomplished.

The relation of microörganisms to suppurative inflammation is one of great importance in its bearing upon the healing of wounds. While not all forms of microörganisms are capable of exciting suppuration—nor are microörganisms the only agents that are competent to excite suppuration—yet the proof is conclusive that the suppurative diseases that complicate wounds, and the acute suppurative inflammations that occur in animals, are caused by the vital activity of various forms of microörganisms that find in the wounds the conditions that favor their development and increase.

The result to be expected in routine manipulations and operations depends very largely upon the practicability to carry out methods by means of which the access of microörganisms to, or their development in wounds, can be prevented or diminished, for where disturbances of repair are escaped the healing is sure, speedy, and perfect. If septic infection could be altogether prevented there would scarcely be any bounds to the possibilities in the domain of veterinary surgery. No decomposition or fermentation takes place in organic matter without the agency of some form of microörganism, and where no extraneous organism has gained access to a wound no wound disturbance occurs. Certain forms of microörganisms are always found associated with certain forms of wound disturbances. Clinical experience shows that where protection from the invasion of microörganisms is secured there is an immunity from wound disturbances.

The recognition of the activity of microörganisms as the essential cause of disturbances of repair in wounds supplies a scientific basis for treatment and affords a definite principle by which to test methods of wound treatment. It is not the organisms themselves that directly cause wound disturbance, but the irritant products formed in the course of their growth and multiplication, either directly secreted by themselves or formed by the decomposition of the substances on which they feed. These secondary products—ptomaïns—are poisons or septic agents, and the results in general of their action upon the living tissue with which they come in contact constitute sepsis. Whatever tissue or wound surface is uncontaminated by these ptomaïns is in an aseptic condition, and whatever method or means antagonizes their production, or antidotes, restricts, or removes the results of their presence is antiseptic.

The ideal treatment of a wound is that by which a perfectly aseptic condition should be obtained and preserved; where this is impracticable the object of treatment becomes changed to the application of means to diminish the activity of the septic organisms, to secure the rapid removal of their products, and to increase the resisting power of the wounded tissues.

Cleanliness is the very first essential in all our routine work—in fact, there is no such thing as asepsis without absolute cleanliness. I do not know how to sufficiently emphasize the importance and actual necessity of cleanliness in each and every detail, if we expect to obtain the best results possible. Practitioners with unkempt surroundings, and who in their actual daily practice belittle their calling by their disregard of water and their careless toleration of dirt, will not be expected, much less inclined, to carry out the scientific principles laid down in this paper. Fortunately this indifferent and careless class of veterinarians have to give way to the more competent and worthy.

(To be continued.)

